# Down syndrome and Alzheimer's disease: common molecular traits beyond the amyloid precursor protein

**DOI:** 10.18632/aging.102677

**Published:** 2020-01-09

**Authors:** Wileidy Gomez, Rodrigo Morales, Vinicius Maracaja-Coutinho, Valentina Parra, Melissa Nassif

**Affiliations:** 1 Laboratory of Neuroprotection and Autophagy, Center for Integrative Biology, Faculty of Science, Universidad Mayor, Santiago, Chile; 2 Departamento de Bioquímica y Biología Molecular and Advanced Center for Chronic Diseases (ACCDiS), Facultad de Ciencias Químicas y Farmacéuticas, Universidad de Chile, Santiago, Chile; 3 Department of Neurology, The University of Texas Health Science Center at Houston, Houston, TX 77030, USA; 4 CIBQA, Universidad Bernardo O’Higgins, Santiago, Chile; 5 Centro de Modelamiento Molecular, Biofísica y Bioinformática (CM2B2), Facultad de Ciencias Químicas y Farmacéuticas, Universidad de Chile, Santiago, Chile; 6 Center for Exercise, Metabolism, and Cancer Studies (CEMC), Facultad de Medicina, Universidad de Chile, Santiago, Chile; 7 Autophagy Research Center, Universidad de Chile, Santiago, Chile; 8 Escuela de Biotecnología, Facultad de Ciencias, Universidad Mayor, Santiago, Chile

**Keywords:** Alzheimer's disease, Down syndrome, mitophagy, epigenetics, oxidative stress

## Abstract

Alzheimer’s disease (AD) is the most prevalent type of dementia. Down syndrome (DS) is the leading genetic risk factor for Early-Onset AD, prematurely presenting the classic pathological features of the brain with AD. Augmented gene dosage, including the *APP* gene, could partially cause this predisposition. Recent works have revealed that alterations in chromosome location due to the extra Chromosome 21, as well as epigenetic modifications, could promote changes in gene expression other than those from Chromosome 21. As a result, similar pathological features and cellular dysfunctions in DS and AD, including impaired autophagy, lysosomal activity, and mitochondrial dysfunction, could be controlled beyond APP overexpression. In this review, we highlight some recent data regarding the origin of the shared features between DS and AD and explore the mechanisms concerning cognitive deficiencies in DS associated with dementia, which could shed some light into the search for new therapeutic targets for AD treatment.

## INTRODUCTION

**Figure 1:**
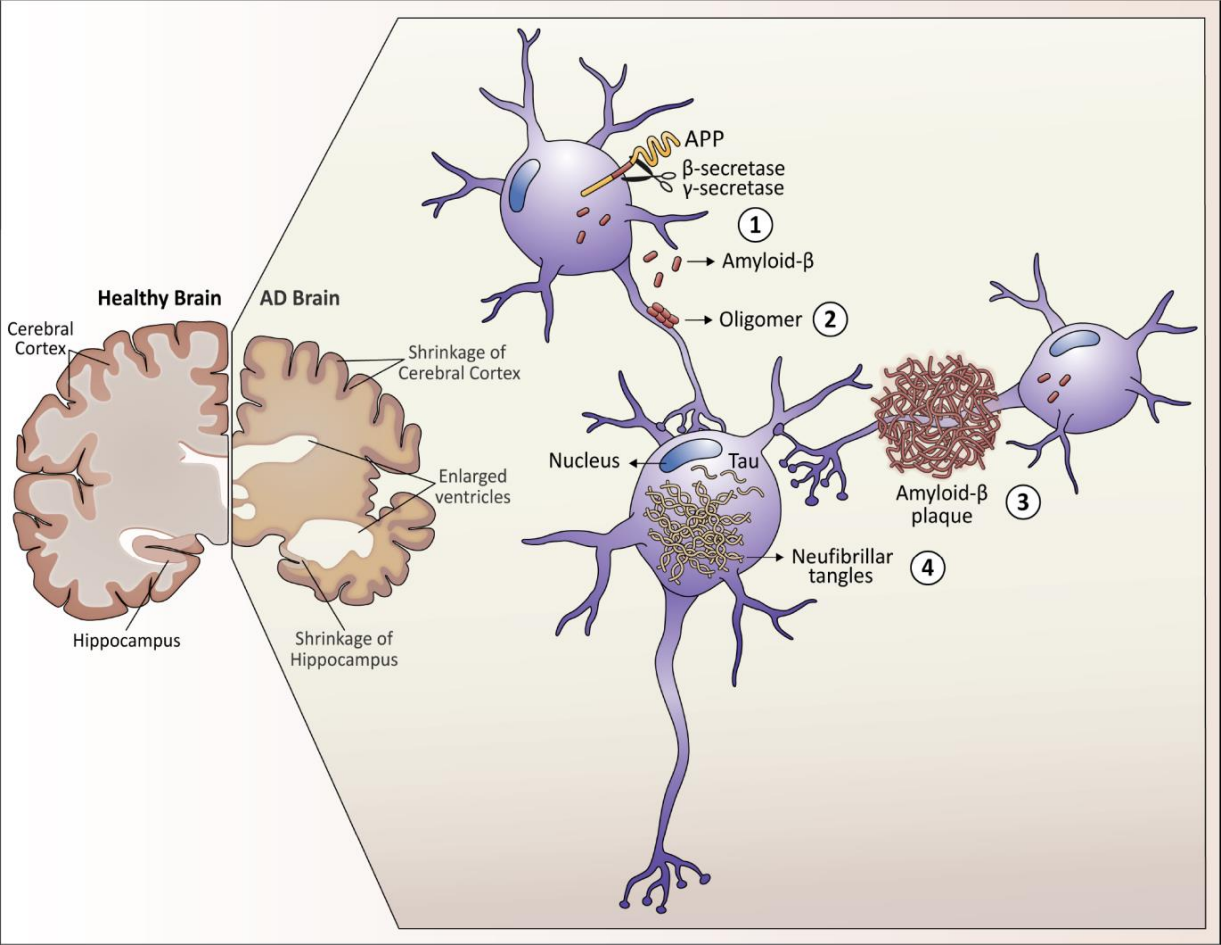
**Neuropathological hallmarks that characterize Alzheimer’s disease.** As Alzheimer's disease progresses, the brain tissue shrinks, the volume of the ventricle, which contains cerebrospinal fluid, increases markedly. At the molecular level: 1. Amyloid-β peptides are produced by the cleavage of the amyloid precursor protein (APP) in the membrane of the neurons. 2. In the space between the neurons, amyloid-β forms oligomers that are thought to disrupt the function of the synapses and act in receptors present in the neuron plasma membrane. 3. The fibrils of the amyloid-β oligomers are added in plaques, which interfere with the function of the neurons. 4. Tau hyperphosphorylation causes neurofibrillary tangles within neurons, displacing intracellular organelles and disrupting vesicular transport.

Alzheimer’s disease (AD) is a progressive disease representing the most prevalent cause of dementia in the elderly, affecting millions of people worldwide. Clinical signs of dementia include a progressive decline in cognition, memory, and language. Specifically, AD is characterized by a loss of short-term memory and other mental abilities, as the neurons responsible for these skills are gradually lost. Almost 70% of the 50 million people that live today with dementia have AD. With the increase in life expectancy in most middle and developed countries, these numbers are expected to rise to 150 million people living with dementia by 2050 [1]. Neuropathological hallmarks that characterize AD are the accumulation of amyloid-β (Aβ) peptide and tau protein hyperphosphorylation, resulting in intracellular neurofibrillary tangles (NFTs) and atrophy of some areas of the brain due to neuronal loss (Figure 1) [2]. Despite that more than 100 years have passed from its first description by Alois Alzheimer, the etiology and sequential pathological mechanisms of AD are still a subject of debate. Moreover, the majority of AD cases are called sporadic or late-onset AD (LOAD), with an unnoticed direct cause. However, we know that the main risk factor for LOAD development is the advance of aging, in parallel with the progressive weakening of homeostatic processes in the organism. Of note, one of the genetic risks correlated to LOAD development includes the variant ε4 for the apolipoprotein E (*APOEε4*) [3, 4], which interferes with Aβ peptide clearance, generating robust Aβ-plaques [5]. About 1 to 5% of AD cases are called early-onset (EOAD), presenting clinical signs before the age of 65. About 5% of EOAD is known to be caused by autosomal mutations, like those located in genes encoding proteins responsible for Aβ generation, such as *APP* (amyloid protein precursor) and *PS1/2* (presenilin 1 and 2, part of gamma-secretase enzymes) [6]. However, most cases of EOAD remain unexplained [6]. Individuals with Down syndrome (DS) represent the largest group of individuals under 65 years of age with EOAD, presenting an early appearance of the three classical features of AD. In that sense, DS is currently considered the leading genetic risk factor for EOAD [7]. In the last decades, the life expectancy of individuals with DS has improved considerably, and, as aging is the primary risk factor of AD, the incidence of mixed pathology in this population has shown a similar trend [7]. This issue is of great concern, since, to date, there are no treatments to delay, stop, or prevent AD. The high incidence of AD in adults with DS, together with the ability to identify these individuals before or during birth, brings opportunities for the discovery of new biomarkers in DS individuals before the appearance of AD-associated clinical signs, as well as a better understanding of the pre-clinical mechanisms related to AD [8]. In the present work, we highlight the molecular crosstalk between DS and AD, and our main focus is discussing novel evidence regarding mitochondrial function and dynamics, as well as molecular and epigenetic regulation, during the progression of AD in DS individuals.

## Neuropathology of Alzheimer's disease

AD is a slowing evolving disorder whose neuropathological features start to appear in the brain about 20 years before the onset of the symptoms [9]. Current AD diagnosis is based on clinical signs and the systematic exclusion of other potential dementias, including other tauopathies or frontotemporal dementia (FTD) [10]. However, and despite the efforts of neurologists, from 10 to 30% of patients diagnosed with AD by clinical symptomatology do not display the AD neuropathological changes characteristic of the disease in *postmortem* analyses [11]. Therefore, AD has been recently defined as a disease that presents progressive neuropathological changes that can be visualized *in vivo* as biomarkers, more than just based on clinical symptoms that are consequences of the disease [2]. The aforementioned neuropathological changes are the i) Aβ plaques deposited in the brain parenchyma and vessels, which can be visualized *in vivo* by positron emission tomography (PET) with specific stains; ii) intracellular deposition of NFTs, also observed by PET; and iii) neurodegeneration, assessed by structural magnetic resonance imaging (MRI) and visualized as the atrophy of specific brain areas [2]. Although there is a consensus about the presence of these biomarkers for definitive AD diagnosis, a direct causality between Aβ production, tau hyperphosphorylation, and neuronal cell death has not been proved [12]. Furthermore, the pathology start point and temporal spreading of both proteins are different: Aβ plaques primarily form in the neocortex and spread to deeper brain areas, while tau starts its accumulation in limbic regions, from where NFTs spread to the neocortex [13–15].

### Amyloid-β plaques

The main component of Aβ plaques in AD is the Aβ peptide. Aβ is derived from the sequential cleavage of APP by *β*-secretase 1 (BACE1) and complex γ-secretases. APP is a transmembrane protein present in the plasma membrane and other organelles of neurons, glia, and other peripheral tissues [16]. This process can produce peptides with 40 or 42 amino acids, called Aβ40- and Aβ42-peptides. The two extra amino acids of the Aβ42-peptide confer the molecule an increased propensity to aggregate, classifying it as the most “amyloidogenic.” This process was better understood when the first case of familial EOAD was elucidated and proven to be caused by mutations in the *APP* gene, resulting in an increased production of Aβ42-peptides [17]. Two other mutations in genes coding for the two components of the γ-secretase complex, *PSEN1* and *PSEN2*, were also associated with familial EOAD [18]. In pathological conditions, Aβ forms aggregates, ranging from soluble oligomers to long amyloid fibers, of which the latter can be visualized by PET and the use of specific radiopharmaceuticals.

Interestingly, despite differences in the onset, the distribution of Aβ accumulation throughout the brain is similar in EOAD and LOAD, affecting brain regions that serve as convergence areas for information originated in different and multiple processing places (called hubs), such as the medial, frontal and parietal cortex [19]. Although Aβ oligomers have been proposed to be more toxic than plaques, both structures are known to cause synaptic impairment and neuroinflammation [20, 21]. However, Aβ accumulation does not directly correlate with neurodegeneration in AD, characterized by neuronal loss and brain atrophy. Likewise, treatment strategies against AD using antibodies targeting Aβ-42 have shown no improvement in cognition in human clinical trials, despite the clearance of Aβ plaques in animals models (discussed in [22]).

One possibility is that other species of APP fragments, different from Aβ fibers, might be involved in neuronal death. With the advent of more specific antibodies, recent work has shown an unexpected accumulation of an APP intracellular fragment generated after β-secretase activity: the C99 fragment [23]. C99 is a C-terminal membrane-associated APP intermediate with 99 amino acids produced before the cleavage of γ-secretase. This fragment is preferentially produced and accumulated in the endosomal autophagic-lysosomal (EAL) system [24, 25], which contains the first organelles affected in AD. Recent studies have shown that C99 exhibits neurotoxic effects independently of Aβ-monomers or oligomers in animal models of AD and fibroblasts from AD patients [23, 26, 27]. Of note, the first site of Aβ accumulation in the central nervous system (CNS) of DS individuals is intracellular [28, 29], occurring in the EAL compartments [30]. Thus, current studies are exploring the effects of APP metabolism products, independently of the extracellular accumulation of Aβ in AD.

On the other hand, the temporal and spatial distribution of NFTs formed by pathological tau modification (hyperphosphorylation) has been more directly correlated to neuronal death and neurodegeneration in AD. However, recent evidence is finally proposing mechanisms for a synergic effect of both proteins in the pathogenesis of this condition [31, 32].

### Neurofibrillary tangles

NFTs are mainly composed of hyperphosphorylated tau protein, which is a microtubule-associated protein highly expressed in the CNS, especially in neuronal axons. In normal conditions, tau stabilizes the cytoskeleton of microtubules, which is essential for cell stability and vesicle trafficking [33]. In humans, the same gene can generate six isoforms of tau by alternative splicing, each of which is classified according to the number of microtubule-binding repeat sequences present in the molecule. In AD, the affected tau isoforms are the ones with three- (3R) and four-repeats (4R) [34, 35]. Under pathological circumstances, soluble tau proteins undergo hyperphosphorylation processes, leading them to adopt anomalous forms and aggregates in insoluble and toxic inclusions -the NFTs- that are mostly located in the neuronal soma. Intracellular consequences of NFTs presence are microtubule disintegration and neuronal communication dysfunction. The latter is caused by the collapse of the transport system, which eventually also causes the activation of cell death [36].

Researches have been exploring the role of both proteins (Tau and APP) in AD pathogenesis for decades. Recent studies are finally proposing an interesting picture, where Aβ plaques would work as a priming factor to tau toxicity in the brains of AD patients. Although both processes start in different brain areas, when NFTs reach the region where Aβ plaques are accumulated, they potentiate tau hyperphosphorylation and neuronal toxicity [31]. In this scenario, Aβ plaques are not responsible for tau modifications and accumulation, as the amyloid theory has tried to explain. Instead, Aβ plaques would create a stabilizing environment for the NFTs, facilitating tau toxicity [37]. This hypothesis would explain how it takes almost 20 years to develop AD before the beginning of the clinical symptoms. Interestingly, DS individuals present both protein phenomena at early ages of their lives.

## Alzheimer's disease pathogenesis in Down syndrome

DS is a genetic disorder caused by the presence of an extra copy of Chr21 or a part of it. It is characterized by a complex and variable phenotype, including craniofacial abnormalities, heart defects, neurological alterations, and cognitive impairments [38]. DS is one of the most studied human syndromes, and DS neuropathology research has become a revisited field during the last decade, for a number of reasons: i) DS is the leading single genetic risk for the development of EOAD [39, 40]; ii) DS has been considered as a human model of accelerated aging, or a model of premature aging (question still under debate); iii) DS allows the correlation between genetic defects and pathological phenotypes; and iv) DS neuropathology is associated with neurogenesis defects, brain development abnormalities, and cognitive impairments [41]. Overall, the neuropathological changes associated with AD in the DS population are characterized by the initial formation of Aβ plaques within the cerebral cortex and then progressing into the hippocampus, striatum, and cerebellum [42, 43]. Moreover, NFTs develop in neurons that project to those areas, presenting a pattern of spread similar to that seen in AD [44]. These and other processes have allowed researchers to consider DS as a model of preclinical AD, thus contributing to the understanding of the pathological mechanisms involved in the progression of this disease [43, 45].

Researchers have identified 233 gene encoders in Chr21 [46]. In addition, RNA molecules that do not translate into a protein (called non-coding RNAs (ncRNAs), associated with gene transcription regulation) are also found in Chr21. A survey that integrates data from GENCODE 31 [47], Ensembl GRCh38.95 [48] and a set of long non-coding RNAs (lncRNA, discussed in detail below) identified by Amaral and colleagues [49], revealed the presence of 1,050 lncRNAs in Chr21, in addition to 30 microRNAs (miRNAs). For this reason, it is possible that other candidate genes or regulatory sequences encoded in Chr21 [50] may interfere with Aβ aggregation and other events, thereby triggering the early onset of AD, beyond *APP*.

To date, evidence indicates that more than 600 genes are overexpressed as a consequence of trisomy 21 [40]. In this regard, *Chou* et al. analyzed differences in the expression of genes that presented only two copies in trisomic and disomic tissues, under the hypothesis that these differences may contribute to the phenotypic variations observed in DS. This work found that several disomic genes present higher expression variances in human trisomic tissues compared to normal ones, and the number of disomic genes with high variance was significantly higher in trisomic tissues versus normal ones. This data suggests that the genetic imbalance observed in DS leads to greater instability in transcriptional control [40, 51].

Another study published in 2014 with discordant monozygotic DS twins analyzed the transcriptome of induced pluripotent stem cells (iPSC) derived from fibroblasts to find chromosomal domains with different expression profiles [52]. In this work, the DNA of the twins showed regions of increased expression, while others showed the opposite behavior. These domains, not considered random regarding their organization, were denominated GEDDs (Gene expression dysregulated domains) [53]. Thus, the organization in GEDDs can be the result of the overexpression of one or more Chr21 genes, which leads to modifications of the chromatin environment. These changes in the nuclear compartment of trisomic cells influence the overall transcriptome. Thus, GEDDs may be an important contributor to the origin and development of DS and AD-associated pathologies in DS individuals [54].

Some other relevant cellular consequences of Chr21 third copy and the associated genes that could promote the AD-like pathology in DS individuals are described below in detail.

### Amyloid plaques

**Figure 2:**
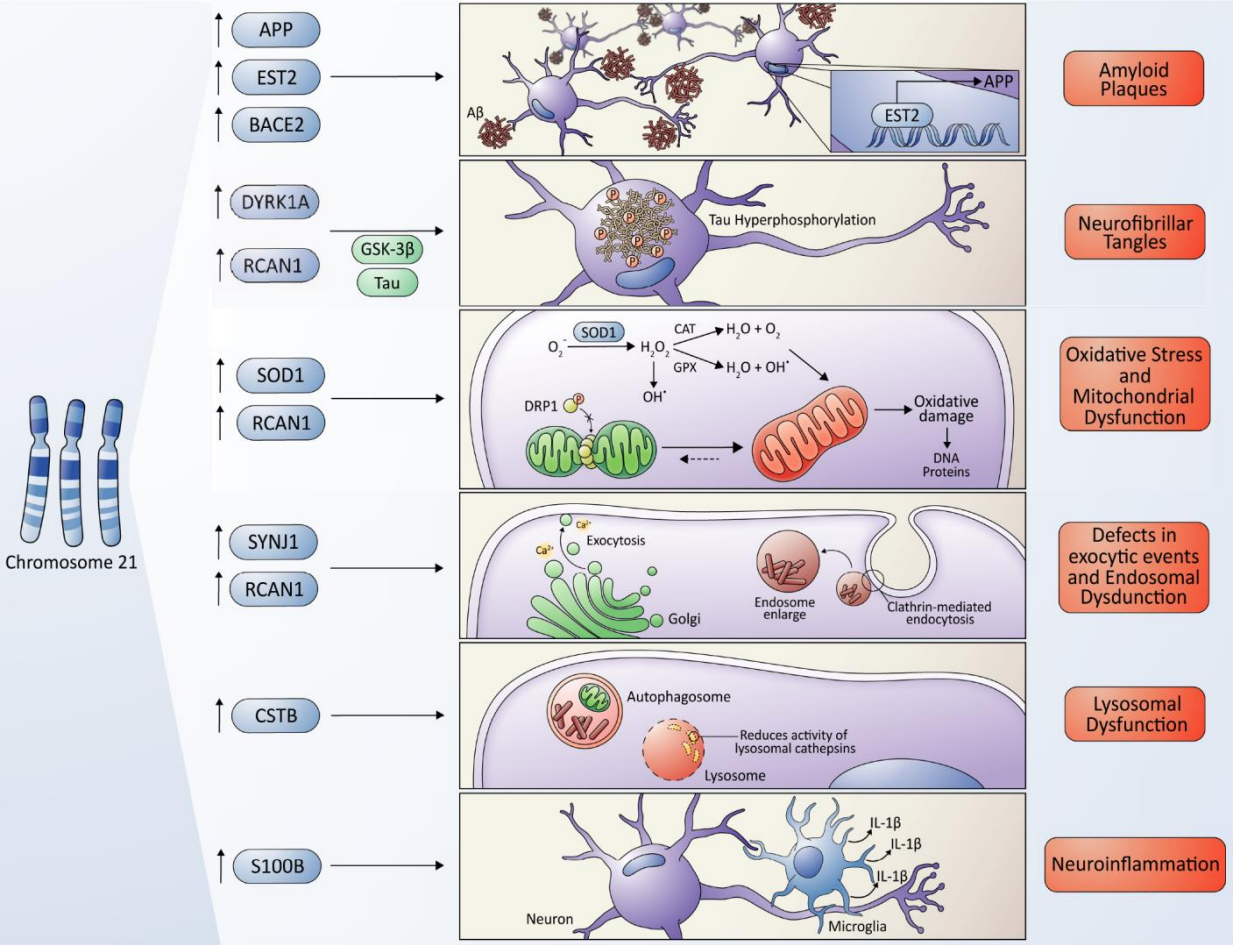
**Molecular cross-talking between Down syndrome (DS) and Alzheimer's disease (AD).** Overexpression of some genes located on chromosome 21 have been linked to the development of neuropathological characteristics of AD in DS some individuals, including the *APP* gene, which encodes the amyloid precursor protein, and the *EST2* gene that encodes a transcription factor that promotes the expression of *APP*, give rise to the Aβ toxic peptides, which form the amyloid plaques. Also, the overexpression of *RCAN1* and its activity as an inhibitor of the phosphatase Calcineurin contributes to the hyperphosphorylation of tau driven by some kinases, among them, the kinase encoded by the *DIRK1A* gene giving originating neurofibrillary tangles. *SOD1* leads to an increase in ROS levels and oxidative stress due to an imbalance in the ratio of SOD1 and other antioxidant enzymes, resulting in a final accumulation of H_2_O_2_, that contributes to mitochondrial dysfunction, producing a higher quantity of ROS, characteristic of both pathologies. However, a protective role of SOD1 in DS/AD was also proposed due to an indirect reduction in Aβ cytotoxicity (please, see details in the main text); the *RCAN1* gene has been linked to the increase in mitochondrial fusion, triggering an elongated mitochondrial network, which in turn increases ROS production. The gene encoding the phosphoinositide phosphatase synaptojanin 1 (*SYNJ1*), a key regulator of the signaling phospholipid phosphatidylinositol-4,5-biphosphate, has been linked to the endosomal dysfunction in DS and AD. Besides, APP processing takes place in the endosome/lysosome system as well. The Cystatin B (*CSTB*) gene functions as an endogenous lysosomal protease inhibitor, and it is found to inhibit the functions of cathepsins, contributing to dysfunction in lysosomal proteolysis. The astrocyte-derived cytokine S100B has been associated with the activation of glial cells, following by an increase in IL-1β in the nervous system, influencing the neuropathology of AD and DS.

The *APP* gene is one of the genes overexpressed in DS (Figure 2) with full trisomy 21 [55]. It encodes the amyloid precursor protein that originates the Aβ-peptide, which is generally observed as diffuse deposits that, as age advances, progress to neuritic plaques, corresponding to one of the pathological hallmarks shared between DS and AD [56, 57]. The gene encoding the ETS Proto-Oncogene 2 (*EST2*) transcription factor (Figure 2) is also located on Chr21 and this factor activates the *APP* promoter, contributing to its overexpression [7]. In addition, overexpression of *ETS2* triggered by chronic oxidative stress and mitochondrial dysfunction, both present in the brains of people with DS and AD, has been associated with neurodegenerative lesions. *Helguera* et al*.* proposed that modulation of *EST2* expression and the preservation of the redox status and mitochondrial function, would be relevant to protect neuronal homeostasis, prevent cognitive deterioration and the development of AD in people with DS [58].

Different studies have shown that the deposition of Aβ can occur from the early age in people with DS, but after age 30-40, they are usually observed systematically, and their accumulation is exponential, meaning not only an early onset of the disease, but also an acceleration in the deposition of Aβ plaques, compared to the general population [59]. Neuropathological findings similar to those observed in AD in people with DS prompted different researchers to answer the question if the overexpression of the *APP* gene was sufficient to promote a pathology similar to AD. Human APP overexpression in mice (a transgenic mouse model of AD, TgCRND8 mice) is sufficient to promote an AD-like pathology that includes Aβ deposition, dystrophic neurites, and learning and memory impairments [60]. Interestingly, these animals also display alterations in the neocortex and hippocampus, mimicking some of the pathological features observed in DS. Likewise, high expression of APP in fibroblasts of individuals with DS is necessary and enough to cause morphological and functional anomalies in early endosomes, which participate in neuron growth, homeostasis, and synaptic functions [61].

At the onset of AD, neuronal endosomes become abnormally enlarged, as in DS, resulting in endosomal dysfunction and neuronal vulnerability [26, 61]. Confirming these results, endocytic abnormalities were reversed by reducing the expression of APP or BACE1 in APP-transgenic mouse models of AD (TgAPP mice) [62]. In addition to Aβ, multiple other APP metabolites are thought to contribute to the neuropathology of DS, as mentioned before. In an elegant work, Wiseman and co-authors generated a DS/AD mouse model by cross-breeding TgAPP mice with trisomic transgenic mice that present the triplication of most genes from human Chr21, except *APP* (Tc21) [63]. Though *APP* is not triplicated, the triplication of other coding genes or ncRNAs from Chr21 was sufficient to increase the soluble Aβ42/Aβ40 ratio and worsen the cognitive decline in the DS/AD model associated with the AD [63]. Although the authors have not shown which genes or ncRNAs are responsible for this phenotype, these results highlight that we have unnoticed the complexity of APP protein metabolism. Clarifying which other factors are acting in this process will provide novel therapeutic targets for AD, in individuals with DS and other conditions.

The β-Secretase 2 (*BACE2*) gene, homolog to the β-secretase of *BACE1*, is also transcribed from Chr21 (Figure 2). However, BACE2 does not exert β-secretase activity and, in fact, cleaves APP on the carboxy-terminal side of the β-secretase cutting site, preventing the generation of Aβ [64]. While *BACE2* mRNA is increased in DS, post-transcriptional regulatory mechanisms can either prevent an increase in translation or a decrease in the rate of mRNA degradation. These findings suggest that *BACE2* is probably not responsible for AD pathology in the DS brain and that it may have a protective function [65]. Nevertheless, a recent study has faced these previous results, suggesting that some AD-associated mutations in *APP* may indeed enable a conditional β-cleavage activity in BACE2, increasing the generation of C99/Aβ progressively in time [65]. Thus, the contribution of BACE2 to the AD pathogenesis might be dependent on the genetic background of patients.

Furthermore, genes located on Chr21 that participate in neuroinflammation also play an essential role in both pathologies. For instance, complement proteins associated with innate immunity have been observed in association with Aβ plaques and dystrophic neurites in both AD and DS brains [59]. Accordingly, individuals with DS have a higher incidence of autoimmune diseases and infections and show brain overregulation of pro-inflammatory markers, including interleukin 1 (IL-1β) [7].

### Neurofibrillary tangles

DS people aged 30–40 show early pathological changes of tau in the outer layer of the hippocampus and, subsequently, NFTs are observed in this structure, together with neural loss in the entorhinal cortex [45]. The NFTs follow a similar pattern of distribution in DS and AD, beginning in the entorhinal cortex and extending towards the hippocampus. However, a higher density of NFTs has been observed in DS brains, compared with AD brains, but a more significant number of studies are needed to confirm these observations [45].

Increased Dual Specificity Tyrosine Phosphorylation Regulated Kinase 1A (*DYRK1A*) gene dosage due to Chr21 trisomy may also contribute to the early onset of neurofibrillary degeneration in DS (Figure 2), through the phosphorylation of alternative splicing factors, that alters the relative abundance of tau protein with three or four microtubule-binding domains [66]. Recent *in vivo* studies performed in animal models overexpressing this gene have demonstrated that *DYRK1A* plays a critical role in several neurodegenerative processes found in DS, including age-dependent cognitive decline, cholinergic neuron degeneration, augmented levels of APP and Aβ, and tau tangles [67]. Accordingly, when the *Dyrk1a* gene dosage is normalized by crossbreeding of a DS mouse model strain (Ts65Dn) with a DYRK1A-KO mouse, the density of the senescent cells in the cortex, hippocampus, and septum is decreased [67]. Moreover, the degeneration of cholinergic neurons is prevented and the expression of APP in the hippocampus, the load of Aβ in the cortex and the hippocampus, and the expression of phosphorylated tau in the hippocampus and cerebellum are reduced [66]. In the same line, other recent evidence has shown that pharmacological inhibition of DYRK1A is sufficient to decrease the Aβ load and insoluble tau accumulation in a mouse model of AD [68].

In parallel, the overexpression of another gene located in Chr21, the regulator of Calcineurin 1gene (*RCAN1*), also called the DS critical region gene 1 (*DSCR1*) (Figure 2), is also implicated in DS tau pathology via stimulation of the GSK-3β (kinase involved in tau hyperphosphorylation) and the inhibition of Calcineurin. Moreover, *Hoeffer* et al*.* also demonstrated that *RCAN1* plays a role in memory and synaptic plasticity by examining the behavioral and electrophysiological properties of *Rcan1* knockout mice tissues. These mice exhibit impairments in spatial memory and learning, reduced associative cued memory, and impaired late-phase long-term potentiation phenotypes, similar to those seen in transgenic mice with increased Calcineurin activity. These findings suggest that *RCAN1* regulates long-term potentiation and memory by inhibiting Calcineurin phosphatase signaling [69]. Additionally, *Hoeffer* et al*.* showed a role of *RCAN1* in the regulation of innate anxiety. *Rcan1* knockout mice displayed reduced anxiety in several tests of unconditioned anxiety. Acute pharmacological inhibition of Calcineurin rescued these deficits, while transgenic overexpression of human *RCAN1* increased anxiety. These results identify *RCAN1* as a mediator of innate emotional states and a possible therapeutic target for anxiety, considering that anxiety is a shared feature between people afflicted by DS and AD [70].

Finally, several reports have indicated that the progression of tau pathology in DS occurs subsequently from the early-life accumulation of intracellular APP metabolites, but on a more rapid timeframe than in AD, possibly due to excessive tau phosphorylation by DYRK1A and RCAN1-gain of function [71].

### Oxidative stress

Another factor associated with the origin of some pathologies related to DS is altered mitochondrial activity, followed by a failure in the detection and implementation of quality control processes. Impairments in mitochondria quality control result in increased reactive oxygen species (ROS) production that triggers oxidative stress and inflammation, and the release of pro-apoptotic factors. Oxidative damage is consistently observed in DS, beginning at a young age and becoming exacerbated with aging and AD progression [41]. Oxidative stress is generated by the imbalance between the formation and elimination of ROS.

In DS patients, the overproduction of ROS is not normally compensated by the physiological neutralizing mechanisms (antioxidants). This fact can be explained by the overexpression of genes from Chr21 involved in the production of these radicals, and the reduction of antioxidant agents. An example of this is the superoxide dismutase enzyme (SOD1), which plays an essential role in the first antioxidant defense line against ROS. SOD1 catalyzes the dismutation of superoxide to molecular oxygen (O_2_) and H_2_O_2_, which can be converted back to water by catalase (CAT) and glutathione peroxidase (GPX). Therefore, *SOD1* overexpression by trisomy 21 leads to an imbalance in the ratio of SOD1, CAT, and GPX, resulting in the accumulation of H_2_O_2_, which is a substrate for other forms of ROS (Figure 2) [72]. However, the role of SOD1 appears to be not so straight forward. For instance, in young adult DS individuals, and just before the onset of dementia, researchers have found low levels of antioxidant enzymes SOD1/GPX activities, which were correlated with the deterioration of cognitive ability [73]. Moreover, in another work, researchers followed the same patients for four years and found that lower SOD1 activity was predictive of a decrease in memory performance over time [74]. Besides, in a study that sought to demonstrate the effect of the host genotype on phenotypes induced by *APP* overexpression using different lines of transgenic mice (C3H/HeJ and B6), it was shown that overexpression of the *SOD1* gene conferred protection against APP-induced premature death [75]. The explanation for this observation is that the neurotoxic effects of Aβ peptides are guided by peroxides, suggesting that at least one route that explains Aβ cytotoxicity is the production of free radicals, causing an increase in the levels of H_2_O_2_ and lipid peroxides in the cell membrane [76]. This link between oxidative damage, neurodegeneration and the protective effect of *SOD1* overexpression in AD pathology was also proposed in an investigation by *Murakami K* et al*.* [77]. In this study, SOD1 deficiency in a transgenic mouse model that overexpresses the *APP* gene (Tg2576) generated an increase in Aβ oligomerization and memory impairment [77]. Thus, the relevance of SOD1 on the AD development in DS and non-DS individuals should be evaluated more concerning its activity than its expression.

Mitochondria, despite being the primary source of cellular energy and the central organelle controlling cellular homeostasis, also represent the primary production source of superoxides as a byproduct of the oxidative phosphorylation process. These ROS can cause oxidative modifications in mitochondrial proteins, lipids, and mitochondrial DNA (mtDNA), increasing mitochondrial dysfunction and triggering the production of more ROS by damaged mitochondria, thus exceeding the capacity of the different cellular antioxidant systems and causing cell death [78]. Several studies have demonstrated that exogenous H_2_O_2_ induces the expression of APP and Aβ aggregation, which is associated with the production of ROS [79–81]. ROS also induce calcium-dependent excitotoxicity and can trigger impaired cellular respiration, together with alterations of the synaptic functions associated with learning and memory [80]. Cells from patients with DS have shown severe alterations in different mitochondrial proteins [82, 83]. These observations have strengthened the idea that throughout the life of a DS subject, their cells are under a permanent oxidative stress hazard [83]. For instance, cortical neurons of DS patients exhibit a high production of intracellular ROS, resulting in the peroxidation of cell membrane lipids, thus compromising neuronal survival [84].

Moreover, another study has also reported mitochondrial dysfunction in DS fibroblasts and mtDNA mutations in brain tissues from DS patients [85]. The specific process of damaged mitochondria clearance is called mitophagy, a selective type of macroautophagy (please, see below). In a recent work from our group, we showed that RCAN1 helps maintain a more fused mitochondrial network by inhibiting the mitochondrial fission process in trisomic iPSC (a human cellular model of DS) from DS patients [86]. RCAN1 inhibits Calcineurin, which dephosphorylates and activates DRP1 (a mitochondrial fission protein), allowing its translocation to the mitochondria to promote mitochondrial fission. In the presence of increased RCAN1 levels, as in cells from DS individuals, fission of the mitochondrial network decreases, and oxygen consumption is increased, which is consistent with previous studies reporting an increase in oxidative stress in DS cells [85]. Therefore, the increased ROS levels observed in cells of patients with DS could be explained by an increase in the dosage of RCAN1. Although fusion and a more continuous mitochondrial morphology have been linked to an increase in O_2_ consumption and higher ATP production, it is important to mention that a sustained mitochondrial fusion could increase the production of ROS and, more importantly, could also increase mitochondrial membrane potential, thus affecting mitochondrial elimination through mitophagy [86].

### Loss of proteostasis: the failure in protein clearance

Similar to other neurodegenerative diseases, such as Parkinson’s and Huntington's diseases, the accumulation of dysfunctional organelles and misfolded proteins is a feature of AD histopathological studies. In general, the accumulation of cytosolic components is a consequence of deficiencies in intracellular degradation systems, resulting in the loss of proteostasis. Autophagy (or self-eating) is one of the essential quality control pathways for the clearance of cytosolic components through their delivery to the lysosome. Three kinds of autophagy processes have been described in mammals that differ in the way the substrate reaches the lysosome: the chaperone-mediated autophagy (CMA), the microautophagy, and the macroautophagy [87]. CMA and microautophagy can degrade soluble cargos, selectively destined to the lysosome or late endosome, respectively. The macroautophagy (hereafter referred to as autophagy), however, is the crucial process responsible for the clearance of insoluble substrates, including organelles, protein aggregates, and pathogens [88]. Through the formation of *de novo* double-membrane vesicles, called autophagosomes, the autophagy pathway isolates the cargo to posteriorly deliver it to the lysosome via vesicle fusion processes, forming the autolysosomes [89]. In AD brains, a remarkable increase in autolysosomes is observed in *postmortem* brain tissues, displaying partially degraded cargos, including mitochondria [90, 91]. In AD brains, it has already been shown that the last step of autophagy is impaired, including lysosome activity. Indeed, mutations in *PS1* associated with EOAD have been linked to an increase in lysosomal pH by a deficient transport of the vacuolar-type H^+^-ATPase complex (vATPase) to the lysosome membrane, resulting in decreased lysosomal activity [92]. Several studies regarding genetic risks for LOAD have found autophagy/lysosomal deficiencies related to AD susceptibility [93]. A recent study using primary human DS fibroblasts reported an early dysfunction in the lysosomal degradative capacity that was dependent on the additional copy of the *APP* gene and, more specifically, on the APP carboxyl fragment terminal (C99) [94]. Researchers found that a moderate increase in C99 levels was sufficient to impair lysosomal function in DS due to an increase in the luminal organelle pH. Remarkable, this effect was molecularly mediated by a direct physical interaction between C99 with the cytosol-exposed domain of vATPase, which was reverted by specifically lowering C99 levels or adding acidic nanoparticles [94]. Given that C99 levels are also increased in AD patients even without *APP* mutations as mentioned above, these findings contribute to understanding early processes underlying lysosome deficits in both diseases.

In subjects with DS and AD, high levels of the mammalian target of rapamycin (*mTOR*) activation, a central inhibitor of the autophagy machinery, contributes to Aβ generation and the formation of NFTs [95–97]. mTOR activation results in a direct decrease in the functionality of the autophagy/lysosome system and, therefore, impairs the Aβ and NFTs clearance. Moreover, a very recent investigation by Bordi and co-authors reported that primary human fibroblasts derived from individuals with DS are mitophagy-deficient, thus leading to the accumulation of damaged mitochondria with a consequent increase in oxidative stress. This finding was associated with two molecular pathway features: i) the deficiency in the activation of the mitophagy pathway dependent upon PINK1/PARKIN and; ii) the suppression of autophagy, due to mTOR hyperactivation [98].

Endolysosomal dysfunction causes alteration of many cellular processes that are essential for neuronal functioning, including protein renewal at synapses, local signaling and also has subsequent effects on the cytoskeleton, protein synthesis, and retrograde signaling. Overexpression of *APP* is also linked to endocytic changes, events observed in both AD and DS. Endocytosis is critical for the transmission and transport of neurotrophic factors in neuron axons, both in retrograde and anterograde directions. The APP protein is processed by the β- and γ-secretases in the cell membrane and endosomes, which are markedly enlarged in the brains of people at the early stages of AD [99]. In patients with DS, a significant increase in the size of endosomes is also observed at the fetal stage (28 weeks of gestation), long before the development of AD. This data suggests an early failure in endosomal trafficking and/or recycling in DS patients, paralleling early events observed in AD [100].

Another gene located on Chr 21 and associated with endosomal dysfunction is the synaptojanin 1 (*SYNJ1*) gene (Figure 2). SYNJ1 is a polyphosphoinositide phosphatase that dephosphorylates phosphatidylinositol-4,5-bisphosphate, which regulates membrane transduction and membrane trafficking in the endocytic pathway at synapses [101]. The SYNJ1 protein is highly enriched in the brain. Specifically, it is located at nerve terminals and associated with synaptic vesicles, coating endocytic intermediates [102]. *Synj1* mutant mice die early after birth, exhibit accumulation of clathrin-coated vesicles at nerve terminals, and increased synaptic depression in the hippocampus [103].

Moreover, the overexpression of the previously mentioned *RCAN1* gene has also been associated with failures in exocytic events, which contributes to the synaptic dysfunction observed in individuals with DS [103]. Using an *in vitro* model of DS (neuronal cell line derived from the mouse cerebral cortex), the work carried out by *Vasquez Navarrete* et al*.* demonstrated the contribution of the overexpression of *RCAN1* in the decrease of the number of exocytic events induced by Ca^2+^ in trisomic cells [104].

Overall, extensive accumulation of ubiquitinated proteins has also been observed in DS brain tissues [93], further suggesting systemic defects in protein quality control and clearance failure by both the proteasomal and lysosomal systems [105]. For instance, previous studies on neuronal cell culture showed that *RCAN1* overexpression leads to mitochondrial degeneration and lower cellular levels of ATP, which, in turn, resulted in a temporary mTOR inhibition [106]. Nevertheless, mTOR basal activity was recovered after 24 h [106]. In DS and AD individuals, however, the chronic overexpression of RCAN1 during a lifetime would trigger cellular deleterious results, as mentioned before, regarding synaptic dysfunctions, increased oxidative stress and NFTs generation. In the case of AD in DS individuals, more detailed studies are required to address the relationship between RCAN1 and mTOR activity during the neurodegeneration process [95, 106].

Finally, the Cystatin B (*CSTB*) gene, also present in Chr21 (Figure 2), functions as an endogenous lysosomal protease inhibitor that inhibits the function of cathepsins (enzymes that degrade proteins), contributing to the dysfunction in lysosomal proteolysis. In a study carried out by *Yang* et al*.*, researchers demonstrated that the deletion of *CSTB* in a transgenic mouse model of AD that overexpresses APP was enough to improve the learning/memory function in APP transgenic mice, resulting in reduced Aβ pathology [107]. Furthermore, in a posterior study, these researchers found that the deletion of *CSTB* in the AD mouse model (TgCRND8) gave rise to a reduction not only of lysosomal proteins, but also of lysosomal lipids [108], like the gangliosides, which also contribute to neurodegeneration in AD [109]. Although this gene was suggested to not be necessary for the DS phenotypes in a mouse model [110], few studies have explored the role of this gene in the AD-linked symptoms observed in DS humans.

### Apolipoprotein E (APOEε4) in DS

The *APOEε4* gene is located on chromosome 19 and is the most substantial genetic risk factor for sporadic AD or LOAD [100]. Studies have indicated that the inheritance of the *APOEε4* allele promotes the earlier appearance of endosomal enlargement at the preclinical stages of AD. Notably, *APOEε4* is a significant factor modulating the severity of the AD phenotype in DS. Several studies have shown that DS patients who carry *APOEε4* exhibit increased risk of AD, earlier dementia onset, and a more significant amyloid load. Considering the shared genetic characteristics between DS and AD, researchers have speculated that the *APOE4* allele accelerates endosomal pathology in both conditions during the early development of AD [111].

### Neuroinflammation

Neuroinflammation refers to an inflammatory process taking place in the nervous system. It can be triggered by persistent systemic inflammation through the delivery of cytokines and other soluble molecules from peripheral immune cells, such as mast cells; or directly, by the engagement of nervous system glial cells, especially microglia. Neuroinflammation participates in the pathogenesis of several neurodegenerative diseases, including AD (reviewed in [111–113]), challenging the view of AD as a neurocentric disease.

Overexpression of the astrocyte-derived cytokine *S100B* (calcium-binding protein B, encoded by a gene in Chr21) and the neuroinflammatory cytokine IL-1β have been identified as early events in DS [114] (Figure 2). *Barger and Harmon* provided the first evidence of a link between neuronal stress, *APP* expression, and neuroinflammation, showing that an elevated release of the α-secretase cleaved fragment, sAPPα (from the non-amyloidogenic pathway), activates microglia and induces the expression of IL-1β, a pro-inflammatory cytokine [115]. According to these results, the search for a relationship between the overexpression of *APP, S100B*, and *IL-1β* resulted in experiments showing that the activation of glia and the resultant increase of IL-1β and S100B in the nervous system influences the neuropathogenesis of both AD and DS. This may be relevant, as the dramatic overexpression of *APP* observed in DS might promote self-propagating cycles of neuroinflammatory cytokines (IL-1β and S100B), an overproduction which in turn, increases *APP* expression [114]. Interestingly, a study evaluating histopathological differences between brain samples from AD-patients and “mismatched” patients (who had no AD symptoms, but whose brains presented Aβ accumulation) showed vast microglial activation in AD, compared to the mismatched samples [116]. Overall, these data suggest a key role for neuroinflammation in AD development.

## Non-coding RNAs and their roles in Alzheimer’s disease and Down syndrome

ncRNAs comprise the most representative transcriptional units of the mammalian genome. Thousands of ncRNAs families have been described, exerting fundamental roles in key molecular mechanisms in organisms from different domains of life [116]. Two of the most studied classes of ncRNAs are the miRNAs and the lncRNAs. miRNAs are part of the large group of small RNAs, which comprise RNA families with 21–25 nucleotides in size [117]. They act as post-transcriptional regulators, leading to degradation or avoiding the translation of messenger RNAs (mRNAs). In contrast, lncRNAs are non-coding transcripts with more than 200 nucleotides in length. Recent research estimates that there are more than 170,000 lncRNAs throughout the human genome. These normally originate from intergenic regions or with some level of overlapping with coding genes. Despite the existence of a large number of lncRNAs in the eukaryotic genome, the mechanism of action of only a small fraction of them is known. Moreover, both miRNAs and lncRNAs are known to be associated with different human diseases, including cancer, cardiovascular conditions, neurological disorders [117], DS [118–120] and AD [121–124].

### MicroRNAs associated with Alzheimer’s disease and Down Syndrome

Thirty miRNAs are transcribed from Chr21 and, therefore, could be potentially overexpressed in DS. Different miRNAs are significantly upregulated in both AD and DS brains, which are known to downregulate the expression of regulatory and anti-inflammatory genes in both diseases [118]. In 2015, *Zhao* and colleagues reported the overexpression of miRNA-155 in the *postmortem* brain tissue of patients with AD, with a potential role in sporadic AD [125]. One of the targets of miR-155 is complement factor H mRNA (CFH), a soluble innate-immune regulatory glycoprotein in AD and DS tissues and in primary brain experimental models of AD, which are also centrally involved in pathogenic signaling pathways that include inflammatory neurodegeneration [126]. More recently, Arena and co-authors described that miR-146a and miR-155 are key regulators of the innate immune response 127. They reported higher levels of miR-146a expression in astroglial cells within the hippocampal white matter of DS, compared with normal fetuses, and identified that this elevated expression persisted postnatally. This may be a key finding, as the expression level of miR-146a has been suggested as an important determinant for neuronal development [115]. In addition, this work revealed the deregulation of these two immunomodulatory miRNAs in an AD mouse model (APP/PS1) and a DS mouse model (Ts65Dn) [128].

Following the overexpression of miR-155 and miR-802 in trisomic iPS-derived neuronal progenitor cells (iPS-NPCs), methyl-CpG binding protein 2 (MeCP2) is degraded. Additionally, trisomic iPS-NPCs exhibited developmental defects and generated fewer neurons than controls. Decreased MeCP2 expression may also contribute to the neurochemical abnormalities observed in the brains of DS individuals [129].

**Figure 3:**
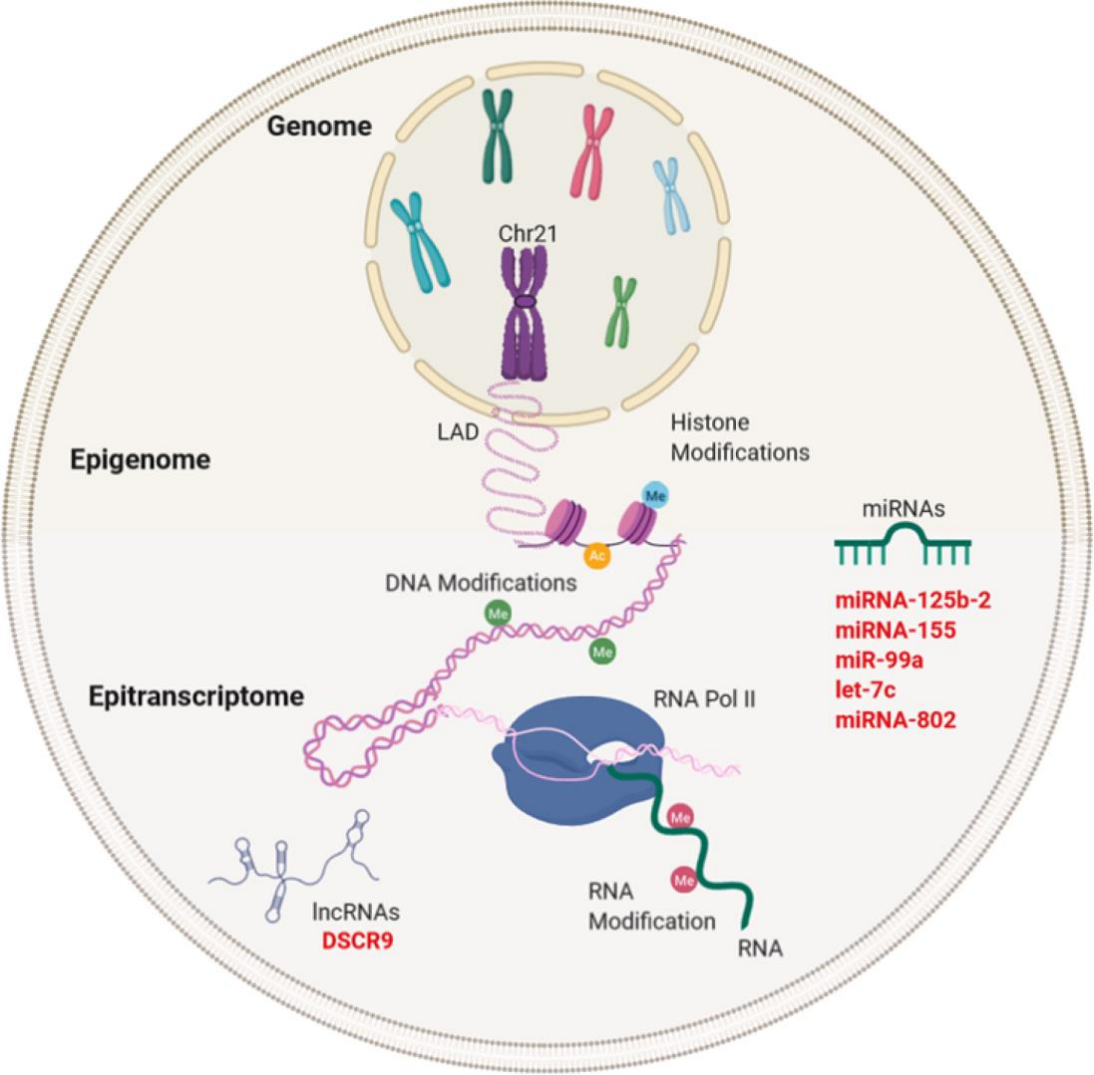
**Gene expression regulation and examples of non-coding RNA in Down syndrome (DS) and Alzheimer's disease (AD).** Gene regulation occurs through the genome, epigenome, and epitranscriptome. Beyond the DNA sequence, chromosomes are regulated by their locations or territories in the nucleus. The presence of an extra chromosome can alter the chromatin structure, ultimately affecting the transcription of the entire genome. At the epigenetic level, gene expression is regulated by reversible modifications of histones within nucleosomes that include methylation, acetylation, phosphorylation, ubiquitination, and sumoylation. Chemical modifications in RNA regulate the fate of transcription through a network of methyltransferases (writers), demethylases (drafts) and specific RNA reading proteins. The regulation of expression by ncRNAs can be affected at different levels. In red letters, the miRNAs and lncRNA encoded in Chr21 linked to DS and AD (miRNA-125b-a, miRNA-155, miR-99a, let-7c, and miRNA-802) and lncRNA (DSCR9) are highlighted.

A computational analysis of the potential miRNAs interacting with synapsin II mRNA, which encodes a neuron-specific neurotransmitter phosphoprotein and is significantly downregulated in AD, showed that it contains 14 potential binding sites for different miRNAs in its 3′UTR mRNA. In addition, synapsin II mRNA has been identified as a target for miR-125b. Small increases in miR-125b expression in AD may have a bearing on synaptic protein deficits, as observed in the AD affected the brain [129] (Figure 3).

### LncRNAs associated with Alzheimer’s disease and Down Syndrome

Even though there is no direct evidence linking lncRNAs to both DS and AD, the presence of more than 1,000 lncRNAs in Chr21, their mechanisms of action, and tissue specificity with higher levels of expression in neuronal tissues may suggest a potential crosstalk between lncRNAs in both diseases. For instance, the expression level of Down Syndrome Critical Region 9 (*DSCR9*) lncRNA, which is transcribed from Chr21, presented a specific tissue expression, and was abundant in the heart and brain, with higher abundance in the hippocampus and amygdala. DSCR9 was highly correlated with genes that were known as important factors in the development and functions of the nervous system, suggesting that DSCR9 can regulate proteins related to DS and other neurological diseases [120] (Figure 3).

To date, some of the lncRNAs associated with AD are *BACE1-Antisense Strand (BACE-AS)* transcribed from the Antisense Strand of the *BACE1* gene. BACE1-AS is capable of overexpressing the *BACE1* mRNA that encodes the protein responsible for the proteolysis of the APP protein [126, 130]. The ncRNA Brain Cytoplasmic RNA 1 (BCYRN1), which modulates the protein synthesis in dendrites, presented higher levels in AD compared to healthy controls, and it was associated with synaptodendritic deterioration [126]. In a study that sought the identification of lncRNAs associated with AD disease using microarray data based on *postmortem* tissue samples from patients with AD and elderly controls, 24 lncRNAs were found to be upregulated and 84 were downregulated in AD patients, compared to healthy controls. Gene set enrichment analysis (GSEA) revealed that the downregulated n341006 lncRNA was associated with the protein ubiquitination pathway, and the significantly upregulated n336934 lncRNA showed to be linked to cholesterol homeostasis, a pathway that has been shown to be dysregulated in AD disease [124].

Finally, a recent study analyzed the RNA expression profile from disomic and trisomic iPSCs [119]. The results showed a significant disturbance in the expression of lncRNAs, compared to protein-coding genes in trisomic iPSC. Moreover, differentially expressed lncRNAs associated with different mitochondrial functions (*e.g.*, mitochondrial organization, electron transport, ATP synthesis, and mitochondrial membrane organization), and most genes related to mitochondria were repressed in trisomic iPSCs, revealing that alterations of lncRNA expression could be related to the mitochondrial dysfunction observed in DS patients [119].

## Epigenetic mechanisms in Alzheimer’s disease and Down syndrome

In addition to the pathological characteristics linked to DS and AD mentioned above, different epigenetic modifications that participate in neurological defects have been identified, mediating brain development and synaptic plasticity [128], further increasing the complexity associated with both pathologies. These epigenetic mechanisms include DNA methylation, chromatin remodeling, nuclear reorganization, but also specific and direct modifications in RNA transcripts, which may influence the final functional roles of different coding and ncRNAs (Figure 3).

### DNA epigenetics

Previous studies have reported that the complete genome has differentially methylated regions, which have been associated with the repression of gene expression, and that are enriched in Chr21 of patients with DS [130, 132]. These results affirm that the presence of an extra chromosome can alter the methylation pattern of the complete genome. At this point, it is convenient to remember that each chromosome occupies a specific place in the nucleus of each cell, and in the case of those cells that have an extra chromosome, the organization of chromatin can vary, thereby altering the expression of the entire genome.

Interestingly, an early onset of epigenetic changes linked to AD and DS pathologies has been suggested. In the work of *Mendioroz* et al*.*, the authors evaluated DNA methylation in the brains of DS fetuses, as well as in the cerebral and cerebellar cortex of adults with DS, and found that some genes that were differentially methylated in fetal brains, kept the same pattern in adult brain cells [131]. This data, along with some other investigations, has been an essential pillar for the future implementation of methylation pattern studies in the prenatal diagnosis of DS, allowing the use of non-invasive techniques with blood samples from the mother [132, 133].

Advances have been made in the development of novel drugs targeting DNA modifying enzymes, called *epidrugs*. Some of these drugs have been successfully tested in AD rodent models, preventing synaptic plasticity disruption and reversing cognitive impairment. This kind of therapeutic intervention emerges as a powerful potential for AD treatment [134, 135].

### RNA epigenetics

RNAs have long been known to contain specific modifications that alter the canonical nucleosides adenosine, cytidine, guanosine, and uridine. More than 170 post-transcriptionally modified nucleosides have been reported in RNA [136]. These post-transcriptional modifications can profoundly influence the structure, stability and base-pairing properties of RNA, and are increasingly recognized as a mechanism for regulating RNA localization, splicing, longevity, interaction with other molecules and RNA base-pairing features [135]. Evidence also indicates that RNA modifications could act as important regulators of synaptic plasticity, memory, and learning [137]. Recent interest has shifted researches to identify modifications in mRNAs, miRNAs, and lncRNAs, driven by advances in next-generation sequencing. These modifications can be achieved by more than 360 different proteins, of which almost 80 of them work as RNA-modifying enzymes (RMEs) in mammals. RMEs comprise three main classes of proteins: "writers," that catalyze reactions; "readers," that recognize the modifications; and "erasers" that remove them [137].

## CONCLUDING REMARKS

Despite the advances towards the understanding of AD pathophysiology, the prevalence of this disorder is increasing enormously worldwide. Currently, no accurate methods of diagnosis, biomarkers, or treatments are available. The current therapeutic approaches that showed positive results in the pre-clinical phase have been disappointing in human clinical trials. They include anticholinergic drugs, antioxidant products, and the last line of biopharmaceutical drugs, monoclonal antibodies against aggregated forms of Aβ. In this regard, it is worth to mention the recent discussion about the human antibody Aducanumab, developed by Biogen Inc. This antibody targets aggregated forms of Aβ, in the hope of reducing its accumulation [138]. After a positive result in preclinical and phase I clinical trials (communicated in 2019), a large phase III trial failed in reducing the cognitive decline in AD patients [139]. Unexpectedly, Biogen announced recently that it will restart the FDA approval process for Aducanumab, indicating that a new analysis of the previous data set showed that the drug reduced the clinical deterioration in early AD patients when it was administered in higher doses [139]. Despite the encouraging statement, a detailed discussion in the scientific and medical field will be necessary when the company finally publishes the complete data [140]. However, and despite this recent achievement, alternative therapeutic targets are required in order to be able to benefit a significant number of individuals from the called “grey tsunami” [141], including DS subjects. DS is the leading genetic risk factor for EOAD development. Thus, an in-depth review of the available information and a thorough analysis of non-conventional genetic data from DS subjects is expected to shed some light on future therapeutic approaches for all cases of AD.

As mentioned earlier, mitochondria have an essential role in cellular homeostasis. Therefore, mitochondrial dysfunction and excessive ROS production can be considered as a convergent mechanism in the neuronal dysfunction associated with DS and AD. Taking this into account, different strategies could be combined to prevent the damage caused by ROS and modulate energetic metabolism, thus avoiding some manifestations of both pathologies [142]. Moreover, the fact that toxic Aβ peptides have been found in the mitochondrial matrix, where they interact with some mitochondrial enzymes, suggests that blocking this interaction could be used as a novel therapeutic strategy to avoid alterations in the Krebs cycle and energy production. Moreover, we also have recently shown that the RCAN1 protein (proven to be elevated in DS and AD), regulates the equilibrium between mitochondrial fission and fusion, increasing mitochondrial ROS production, as well [86], which is a characteristic feature for both conditions. Indeed, in a trisomic model of DS iPSCs, we demonstrated that a decrease of RCAN1 levels was sufficient to rescue mitochondrial morphology and oxygen consumption levels. In this regard, the use of human patient-derived iPSCs proved to be a useful tool for *in vitro* studies, maintaining the unique cellular characteristics that have started to be considered as important hallmarks of both conditions.

On the other hand, increase in epigenomic research and next-generation sequencing has opened new therapeutic options for several diseases, including neurological and genetic disorders. Learning and memory, which are altered in most individuals with DS over 40 years of age, can be modulated by epigenetic mechanisms [143]. Epigenetic markers are reversible, offering an enormous therapeutic potential to alleviate or cure specific genetic deficits. The use of drugs against epigenetic enzymes, or epidrugs, has now emerged as an alternative treatment or could synergistically act with classical pharmacology. More current epigenetic therapies have already been used for cancer and epilepsy [143], and could also provide new possibilities for the treatment of DS and AD to improve cognition. Additionally, DNA methylation provides a new diagnostic method for the detection of DS and novel therapeutic targets that could appear from the investigation of this topic [144].

To date, different therapeutic strategies have been proposed to prevent or stop AD without success, increasing the need for new avenues in dementia research. Exploring the mechanisms concerning cognitive deficiencies in a disease model such as DS could shed some future light into the search for new therapeutic targets for such a devastating disease. In this aspect, in this review, we propose organelles and protein quality control mechanisms, as well as epigenomic research, as two of the most promising fields of study in future AD research.

While protein dosage is an expected cause of AD pathology in DS, it is essential to consider the imbalance in non-coding DNA sequences, and small and lncRNAs present in the context of the trisomy. Although these species are not translated into proteins, they are potent epigenetic regulators of gene expression both in genes located on Chr21 (cis-regulation), and others located on other chromosomes (trans-regulation) [143]. Nevertheless, little is known about non-coding sequences located on Chr21, or in other chromosomes related to pathological aspects of DS. Accordingly, along with deepening the knowledge about the role of these nucleic acid structures in DS, novel biomarkers for early diagnosis of AD could be elucidated. These new biomarkers would benefit not only the diagnosis of AD pathology in individuals with DS but could also offer the possibility of tracking the progression of the disease in its initial stages and monitor the effectiveness of different treatments in the general population [8]. In the present review, we have highlighted the latest findings regarding the common molecular pathways between DS and AD, seeking to emphasize less studied aspects, such as mitochondrial function and epigenetic regulation.
